# Voluntary Medical Male Circumcision: Matching Demand and Supply with Quality and Efficiency in a High-Volume Campaign in Iringa Region, Tanzania

**DOI:** 10.1371/journal.pmed.1001131

**Published:** 2011-11-29

**Authors:** Hally R. Mahler, Baldwin Kileo, Kelly Curran, Marya Plotkin, Tigistu Adamu, Augustino Hellar, Sifuni Koshuma, Simeon Nyabenda, Michael Machaku, Mainza Lukobo-Durrell, Delivette Castor, Emmanuel Njeuhmeli, Bennett Fimbo

**Affiliations:** 1 Jhpiego/Tanzania, Dar es Salaam, Tanzania; 2 Jhpiego, Baltimore, Maryland, United States of America; 3 Ministry of Health, Iringa Region, Tanzania; 4 Office of HIV and AIDS, United States Agency for International Development, Washington, District of Columbia, United States of America; 5 Ministry of Health and Social Welfare, Dar es Salaam, Tanzania; Centers for Disease Control and Prevention, United States of America

## Abstract

The government of Tanzania has adopted voluntary medical male circumcision (VMMC) as an important component of its national HIV prevention strategy and is scaling up VMMC in eight regions nationwide, with the goal of reaching 2.8 million uncircumcised men by 2015. In a 2010 campaign lasting six weeks, five health facilities in Tanzania's Iringa Region performed 10,352 VMMCs, which exceeded the campaign's target by 72%, with an adverse event (AE) rate of 1%. HIV testing was almost universal during the campaign. Through the adoption of approaches designed to improve clinical efficiency—including the use of the forceps-guided surgical method, the use of multiple beds in an assembly line by surgical teams, and task shifting and task sharing—the campaign matched the supply of VMMC services with demand. Community mobilization and bringing client preparation tasks (such as counseling, testing, and client scheduling) out of the facility and into the community helped to generate demand. This case study suggests that a campaign approach can be used to provide high-volume quality VMMC services without compromising client safety, and provides a model for matching supply and demand for VMMC services in other settings.

## Abstract 2

Hally Mahler and colleagues evaluate a six-week voluntary medical male circumcision campaign in Iringa province of Tanzania, providing a model for matching supply with demand for services and showing that high-volume circumcisions can be performed without compromising client safety.

**Summary Points**

- The government of Tanzania has adopted voluntary medical male circumcision (VMMC) as an important component of its HIV prevention strategy and aims to reach 2.8 million uncircumcised men within the next three years.
- In June and July 2010, a six-week VMMC campaign in Tanzania's Iringa Region performed 10,352 circumcisions.
- Strategies adopted by the campaign to generate demand included the widespread dissemination of messages focused on the provision of free VMMC by specially trained health care providers and on the HIV prevention benefits of VMMC.
- Clinical efficiency was improved through, for example, the use of multiple beds in an assembly line, and the efficient use of staff time through task shifting and task sharing.
- The experiences of this campaign suggest that high-volume VMMC can be performed without compromising client safety, and provide a model for matching supply and demand for VMMC services elsewhere.

## Introduction

Several randomized controlled trials have demonstrated the safety and efficacy of voluntary medical male circumcision (VMMC) in HIV transmission prevention among heterosexual men [1]–[3]. Consequently, in 2007, the World Health Organization (WHO) and the Joint United Nations Programme on HIV/AIDS (UNAIDS) recommended that countries with a high prevalence of HIV and a low prevalence of male circumcision scale up VMMC within their comprehensive HIV prevention programming [4]. WHO currently recommends a minimum package for VMMC services that includes group and individual education on VMMC, HIV risk reduction, and other male sexual and reproductive health issues; condom promotion and provision; HIV testing and screening; treatment of sexually transmitted infections (STIs) and physiological abnormalities; and provision of VMMC under local anesthesia with postoperative observation and two to three postoperative visits (two, seven, and 42 days postoperatively) [5]. WHO guidance on optimizing the volume and efficiency of VMMC services [6] recommends the use of multiple surgical beds per surgical team, “task shifting” the surgical procedure from surgeon to nurses and/or clinical officers, “task sharing” less complex steps of the circumcision procedure to lower credentialed but highly trained health care cadres, and the use of forceps-guided surgical techniques, alcohol gel scrubs between surgical cases, and electrocautery for hemostasis.

In 2009, the government of Tanzania adopted WHO's recommendation to scale up VMMC [4]. Tanzania has adult HIV and male circumcision prevalences of 5.7% and 67%, respectively [7]. Regional variations in both male circumcision and HIV prevalence exist that correlate inversely. Religious, educational, ethnic, and cultural factors and differences in the proportion of people living in urban and rural settings may explain some of the regional differences in male circumcision coverage. Notably, a national situation assessment on VMMC reported that 93% of respondents in traditionally non-circumcising areas of Tanzania would take their sons to be circumcised if the services were available, and identified the major deterrents to seeking VMMC as financial costs and having no family or cultural history of circumcision [8].

In 2010, the government of Tanzania set a goal of 80% VMMC coverage in its draft proposal “National Strategy for Scaling Up Male Circumcision for HIV Prevention” [9]. To achieve this goal, approximately four million circumcisions must be completed in the next five years, 2.8 million of them during the initial three-year implementation stage. The Tanzanian strategy for VMMC scale-up prioritizes eight regions of relatively high HIV and low male circumcision prevalence, with men aged 10–24 and 25–34 years as the primary and secondary priority groups, respectively. The strategy permits task shifting and specifies that VMMC services should be free of charge within the public sector.

Iringa Region, a largely rural region with a population of 1.9 million, has the highest adult HIV prevalence in Tanzania (15.7%) and relatively low circumcision coverage (29%) [7]. As part of the national strategy, the five-year target for VMMC in Iringa Region is 264,990 circumcisions [9]; modeling estimates suggest that one HIV infection will be averted for every 4.5 VMMCs performed in this region [10]. Iringa Region was one of the first Tanzanian regions to adopt VMMC as a part of its HIV prevention program [11]. The Iringa regional health authorities, in collaboration with the United States President's Emergency Plan for AIDS Relief (PEPFAR) and the Maternal and Child Health Integrated Program (MCHIP), a project funded by the United States Agency for International Development (USAID) and managed by Jhpiego (an affiliate of Johns Hopkins University), launched the region's VMMC program in September 2009. By April 2010, five health facilities were providing routine, closely monitored VMMC services that covered three of the region's eight districts.

In June 2010, the Iringa Region VMMC program undertook its first VMMC campaign. In this case study, we describe the campaign's approach to service delivery and the factors that influenced its quality, efficiency, and safety, and we discuss how the experiences of this campaign might serve as a model for future VMMC campaigns in the Iringa Region and elsewhere.

## Description of the Campaign

The Iringa Region VMMC campaign was conducted in three districts of the region between June 21 and July 31, 2010, to coincide with school leave, the end of the harvest season, and Iringa's cool season (previous formative assessment revealed strong community preference for VMMC during the cool season [12]). VMMC services were provided Monday to Friday from 8:00 to 17:00, and Saturdays from 8:00 to 13:00, and were coordinated by committees and teams acting at regional, district, and site levels.

At the regional level, a committee of key stakeholders, which was formed three months before the campaign and included regional health and administrative authorities, campaign managers, and monitoring and evaluation experts, provided campaign oversight. The committee supervised VMMC facilities, provided quality assurance of services, and oversaw the dispensing of regular supplies of HIV test kits, condoms, and other consumable commodities such as sutures, gloves, lidocaine, and antibiotics provided by MCHIP. MCHIP also provided additional surgical beds and instruments, and other infrastructure items to the facilities providing VMMC services during the campaign.

At the district level, demand creation subcommittees were composed of district officials, health facility staff, and international and community-based organizations. These subcommittees, which were tasked with recruiting clients and sensitizing community political and administrative leaders, met several times before and during the VMMC campaign.

Finally, each campaign site had a management team composed of the health facility's medical officer in charge, who was responsible for overall service delivery and quality at the site, and a site campaign manager, who was responsible for daily reporting on and oversight of the VMMC service. Every evening during the campaign, site managers participated in a debriefing session with campaign headquarters, during which problems with campaign implementation, shortages of commodities, issues related to client demand, and AEs were addressed.

These three levels of organization worked together to ensure the efficiency, quality, and safety of the campaign. In particular, as described below, they followed the guidance provided by WHO for using human resources efficiently, for maximizing the throughput of clients, for detecting AEs, and for creating a minimum VMMC package [5],[6]. Demand creation innovations were added to this package by MCHIP to match supply with demand.

### Human Resources

The regional campaign committee developed a human resources plan that considered the WHO guidance for improving efficiencies, the number of surgical bays available, the expected client load, and available counseling and clinical staff. In this assembly-line service model, every four beds at a site required one circumcising surgeon, four bed nurses, one or two equipment and commodities runners, an equipment cleaner, and two HIV counselors. Regardless of the number of beds, each site also required a receptionist, an autoclave operator, a janitor, and a data manager. To reduce burnout, the clinical staff on the VMMC team, who were dedicated fully to VMMC throughout the campaign, rotated between roles (surgeon, bed nurse, and counselor). In addition, the most productive “surgeons” rotated among sites to encourage a spirit of collegiality. Other motivators for providers included T-shirts, daily meals, and overtime pay equivalent to US$7.00 per day. The Ministry of Health and Social Welfare paid staff salaries, with the exception of six counselors working for local non-governmental organizations, who were funded through other PEPFAR-supported programs. Finally, to ensure efficient and high-quality service provision, all the nurses (who constituted 80% of the campaign's VMMC providers, including “surgeons”), clinical officers, and physicians involved in the campaign were trained using the WHO/UNAIDS/Jhpiego male circumcision manual [5], and were provided with comprehensive on-site mentoring by a proficient VMMC physician.

### Demand Creation

Several approaches were taken to create demand for VMMC during the campaign. Through the district-based demand creation subcommittees, community-based organizations received a one-day training session designed to enable the promotion of the campaign by peer educators and outreach workers through activities such as traditional theater, small group sessions, one-to-one peer education, and speeches to community groups. These community workers received no additional compensation or incentive for adding the topic of VMMC to their usual activities. Brochures and other print materials were distributed that targeted specific audiences including adolescents and their guardians, female partners of potential VMMC clients, and men aged 18 years and above. In addition, three radio advertisements promoting the campaign ran eight times per day on regional radio stations, and regional officials appeared on local chat and health-related programs in the weeks leading up to the campaign. Finally, during the campaign, the regional health authorities arranged for announcements about the availability of services to be made across facility catchment areas. Based on the national situation assessment, in which cost was identified as a barrier to services [8], the demand creation messages emphasized that VMMC services were now free and would be performed by specially trained health care providers. Additional messages focused on the HIV prevention benefits of VMMC. Demand creation activities were scaled back by the fourth week of the campaign because of overwhelming demand, and midway through the campaign all sites began scheduling clients 1–2 weeks in advance, except for clients traveling long distances, who were given same-day services.

### Increasing Client Volume

**Figure 1:**
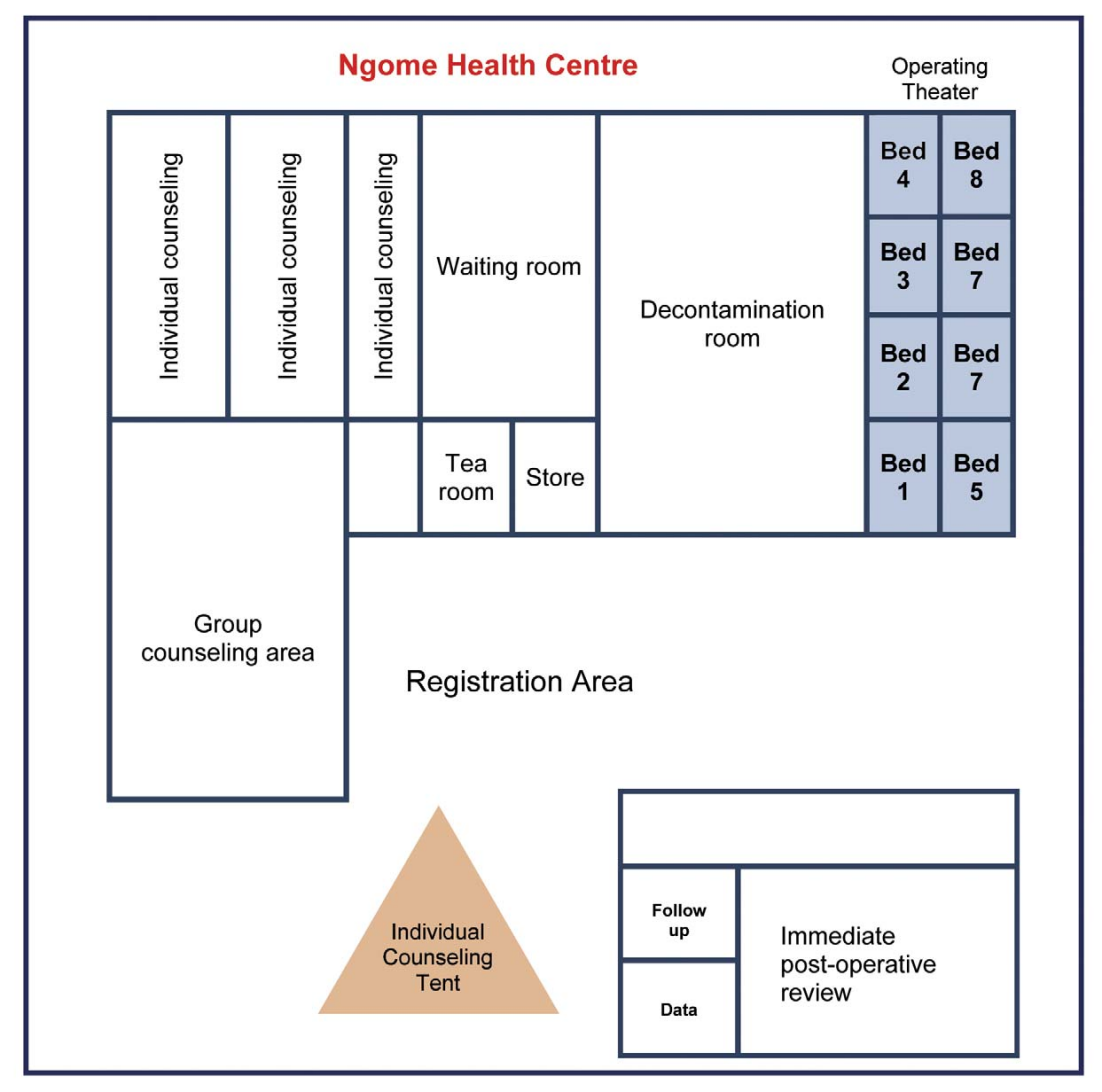
The layout of Ngome Health Centre. Before the campaign, this space was empty, having been built but not yet configured to function as a reproductive health facility. The Iringa Region team adapted the space for efficient VMMC service delivery by expanding the number of surgical bays (eight beds to accommodate two surgical teams), providing a large space for decontamination, increasing the number of individual counseling areas (including a tent to accommodate additional counselors), and including a separate postoperative area.

All of the efficiency considerations recommended by WHO were adopted for increasing client volume, with the exception of electrocautery (which was not endorsed for VMMC services in Tanzania) and the use of disposable surgical instruments kits. Specifically, time-saving surgical techniques, such as the forceps-guided method of circumcision, were used, and the number of surgical kits was doubled at sites without an autoclave to minimize the interruption of services caused by transfer of kits to autoclave-equipped sites for processing. To reduce client congestion during initial and follow-up visits and increase efficiency, the campaign provided additional trained counselors to prepare a large number of clients for the surgery; added tents and other temporary structures at VMMC sites (Figure 1) to create additional space for counseling, postoperative follow-up, and data entry; scheduled clients up to two weeks in advance of surgery; paid special attention to maintaining the motivation of participating health care workers; and developed campaign-specific data monitoring tools.

### Quality and Safety

As stipulated in WHO guidance, HIV testing was offered to all clients on an opt-out basis during individual counseling. Consent for HIV testing was verbal (the standard of care in Tanzania); a guardian's consent was required for clients under the age of 18. VMMC clients who tested HIV-positive received circumcision if they were eligible for surgery (eligibility was based upon their overall health and the absence of physiological abnormalities of the penis or STIs that would otherwise preclude them) and gave consent (during the campaign, all clients provided written consent for surgery), but were counseled about the lack of HIV prevention benefits for HIV-infected men and the increased risk of transmitting HIV to sexual partners during postoperative healing. All HIV-positive clients were referred to HIV care and treatment services, which were available at each circumcising site; clients with STIs were referred to STI treatment services at the same health facility and counseled to return for circumcision after their treatment was complete; and clients with physiological abnormalities were referred to the district or regional urologist.

Infection prevention quality standards were applied at each of the campaign facilities, and the definitions for AEs in the WHO/UNAIDS/Jhpiego “Manual for Male Circumcision under Local Anaesthesia” were used to monitor AEs during the campaign [5] During counseling, the VMMC team emphasized the importance of clients returning for their postoperative reviews, AEs were described to clients to encourage reporting, and each client received details of a 24-hour emergency phone number staffed by VMMC team clinicians. All AEs were recorded using the standard WHO form [5] and were discussed during the site managers' nightly debriefs. The regional committee received weekly briefings on the occurrence of severe AEs and looked for trends to determine whether there might be cause for concern at any one site.

Throughout the campaign, client-level data on service delivery and AEs were entered daily by data clerks within each health facility team into an electronic web-based data system designed for intensive monitoring and feedback. This system allowed each site management team to receive nightly reports of clients prepared, clients circumcised, AEs, and other key client data. The Johns Hopkins University Institutional Review Board and the Tanzanian Ministry of Health and Social Welfare approved the use of these data for this case study.

## Achievements of the Campaign

**Figure 2:**
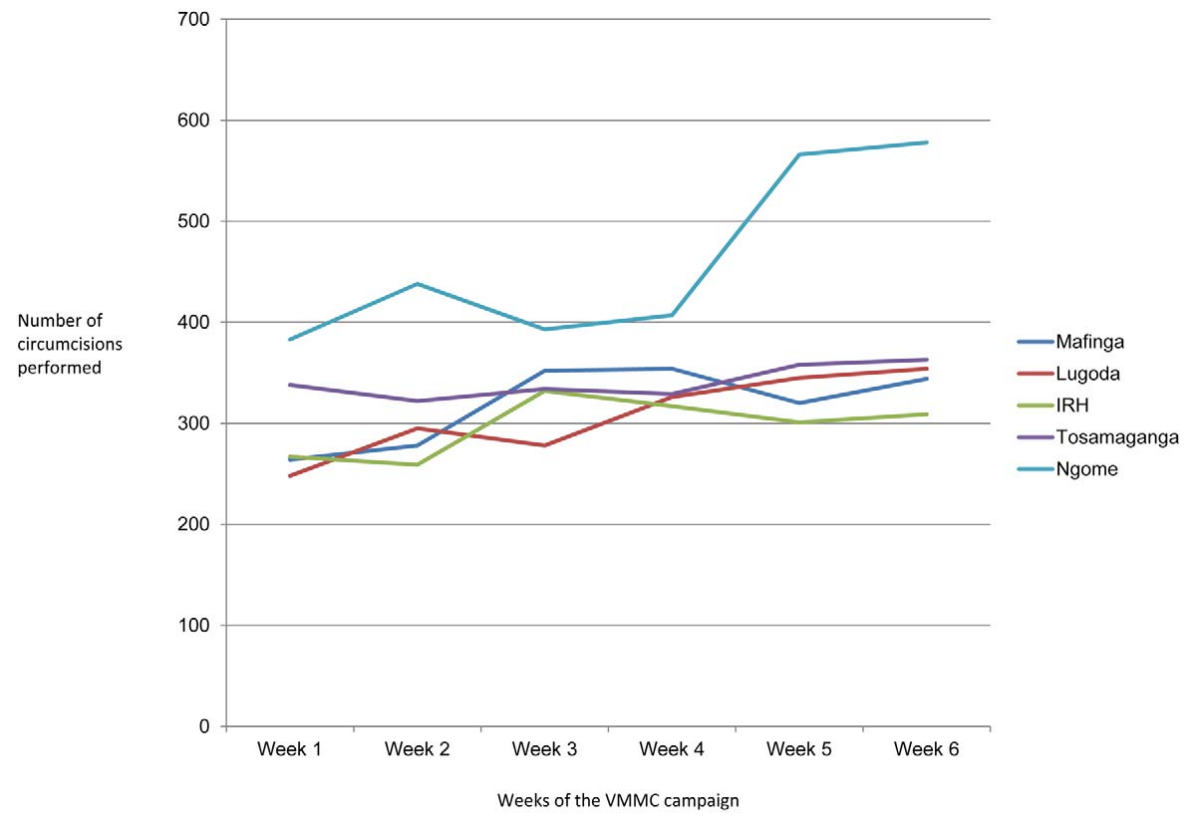
Site-specific upward trends in VMMC service delivery during the campaign. The substantial increase in VMMC delivery at Ngome Health Centre (uppermost line) was due to the addition of more surgical bays mid-campaign. IRH, Iringa Regional Hospital.

**Figure 3:**
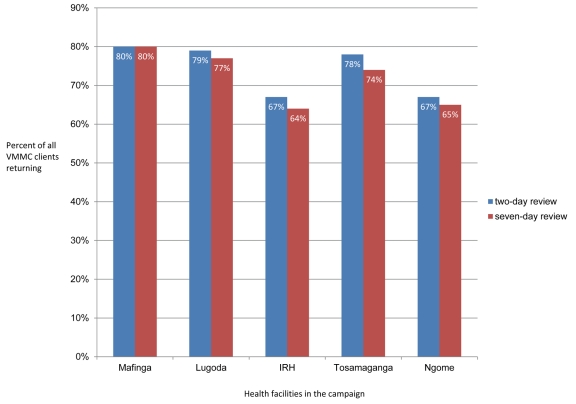
Postoperative return rates two and seven days after surgery.

**Figure 4:**
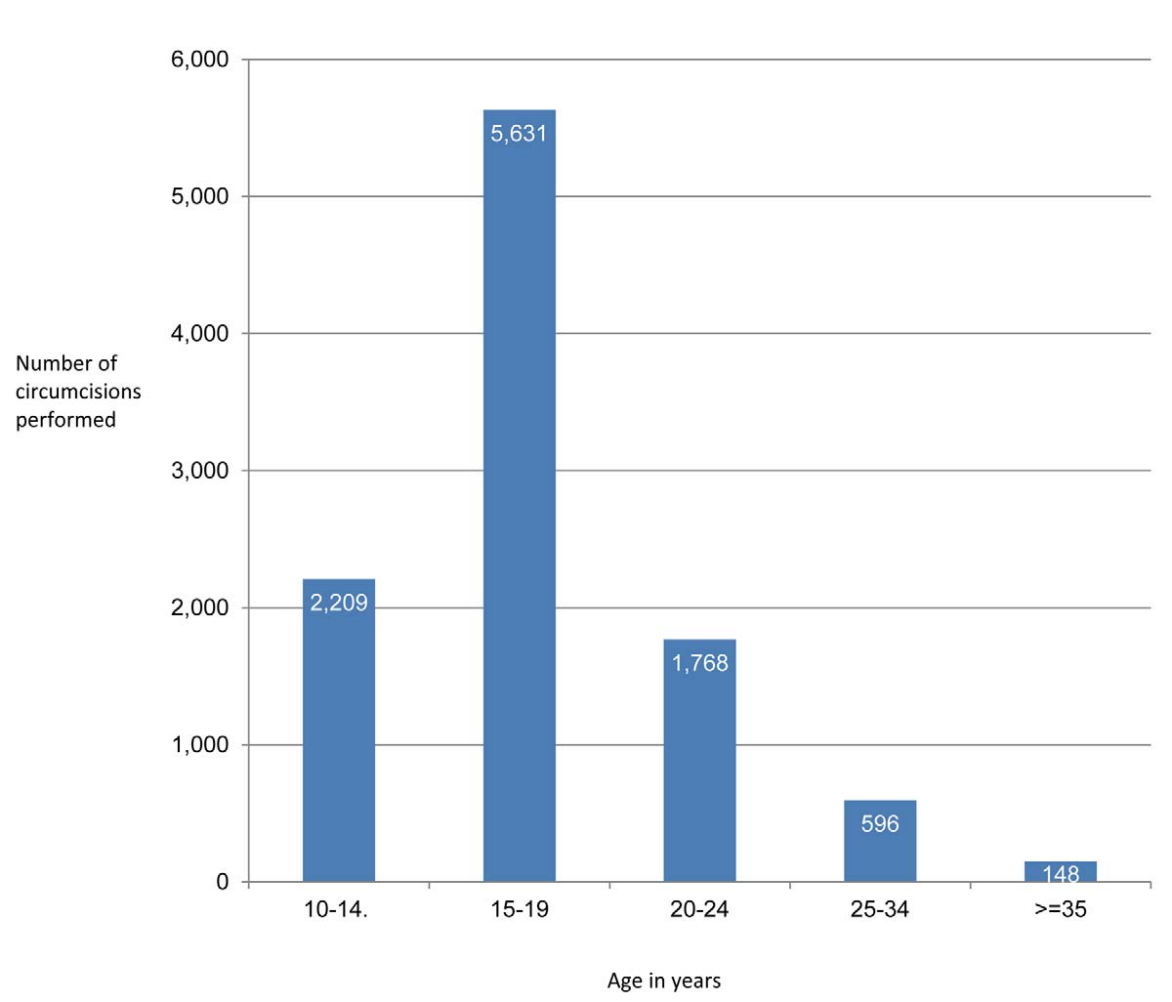
Age distribution of VMMC clients.

During the six weeks of the VMMC campaign, six circumcising teams of 16 individuals, plus site managers, drivers, and data clerks (140 participants in total) circumcised 10,352 adolescent and adult males, 1.72 times the campaign's target of 6,000 men. The average number of clients served per week increased as the campaign progressed (Figure 2), and all the VMMC sites served more than 1,700 clients during the campaign period; Ngome Health Centre, which dedicated a large space to the VMMC campaign, achieved 2,781 circumcisions with eight beds and two surgical teams (Table 1). Staff retention was high throughout the campaign. Overall, 50% of clients came from outside facility catchment areas (defined as further than 15 km from the VMMC site), which suggests that clients were willing to travel great distances for free and safe services. However, there was substantial variability across sites: the percentage of clients coming from outside facility catchment areas ranged from 0.3% at Mafinga District Hospital to 68% at Tosamaganga Hospital (Table 1). Postoperative return rates ranged from 67% to 80% of clients for the two-day follow-up, and from 64% to 77% of clients for the seven-day follow-up (Figure 3). Finally, almost 93% of the clients reached during the campaign were between 10 and 24 years old (Figure 4). Lugoda Hospital, which is on a tea plantation, had the highest percentage of clients in the 20- to 34-year-old age range (43%), presumably because of the large population of adult tea workers in its catchment area.

**Table 1:** VMMCs performed during the campaign, by site.

Site	District	Characteristics of Site	Number of Beds	Number of Surgical Teams	Total VMMCs	VMMC Clients from Outside the Catchment Area^a^	
						*N*	Percent
Iringa Regional Hospital	Iringa Municipal	Urban; regional referral hospital	4	1	1,784	1,114	62%
Ngome Health Centre	Iringa Municipal	Urban; collaboration with Iringa Regional Hospital to serve overflow of clients	8	2	2,781	843	46%
Lugoda Hospital	Mufindi	Rural; services aimed primarily at tea plantation workers	4	1	1,847	6	0.3%
Mafinga District Hospital	Mufindi	Peri-urban; district referral hospital	5	1^b^	1,896	1,874	33%
Tosamaganga Hospital	Iringa Rural	Rural; large, well-established, and well-utilized facility	4	1	2,044	1,394	68%
**Total**	**—**	**—**	**25**	**6**	**10,352**	**5,231**	**50%**

a Catchment area defined as within 15 km of the facility.

b With extra nurse.

The overall AE rate during the campaign was less than 1% (Table 2). Eighty percent of AEs were minor or moderate; only 20% were categorized as severe. The most common intra-operative AE was bleeding; swelling and infection were the most frequent postoperative AEs. All AEs were treated and clients were healed or healing by the end of the campaign.

**Table 2:** Adverse events during the campaign.

AE	Occurrence		Severity		Total AEs
	Intra-operative	Postoperative	Mild/Moderate	Severe	
Damage to penis	1			1	1
Excess skin removal	3			3	3
Excessive bleeding	4			4	4
Swelling of the penis or scrotum		28	15	13	28
Infection		64	64		64
Total	8	92	79	21	100

HIV testing uptake was virtually universal (99%), and the overall HIV prevalence was less than 1% for the campaign clients, although it increased with age (Table 3). Of the 74 individuals who tested HIV-positive, all went on to be circumcised. Twenty-two STI cases were identified during the campaign; all clients with STIs were referred for treatment and subsequently circumcised.

**Table 3:** HIV testing, by age group.

Result	Age				
	10–14 y	15–19 y	20–24 y	25–34 y	≥35 y
Negative	2,199	5,616	1,754	565	134
Positive	10	10	9	31	14
Not tested	0	5	5	0	0
Total	2,209	5,631	1,768	596	148

## Lessons Learned

### Efficiency

The data collected during the Iringa Region campaign indicate that, using the efficiency model adopted by the campaign, a four-bed/one surgeon facility can circumcise up to 60 clients and an eight-bed/two surgeon facility can achieve 120 circumcisions per day over a six-week period, and that the efficiency of VMMC service provision can increase over time. Importantly, the experiences gained during this campaign indicate that, by transferring some VMMC providers from larger sites to smaller sites, it is possible to provide a high-volume service at small sites without greatly impacting on the provision of normal health services provided at these sites. The data presented in this case study also suggest that supply and demand for VMMC can be matched by focusing on community-driven demand, and by ensuring efficient site-level client flow by adding counselors as needed and by expanding the space available for VMMC with tents, careful scheduling, detailed logistics, and the adoption of surgical efficiencies.

### Quality

The long distances traveled by many clients to receive services during the campaign (some clients stated that they traveled as far as 100 km) was unanticipated and suggests that a well-motivated population will travel long distances to VMMC sites during campaigns. However, the willingness to travel long distances may also reflect a need for anonymity or client perceptions of service quality. For example, Iringa Regional Hospital's high “long-distance” caseload may have been related to client perceptions that a referral facility would provide higher quality services, a possibility that warrants further investigation. The high uptake of HIV testing seen during the campaign is not unusual—HIV testing acceptability is generally very high in Tanzania—but may have been magnified by a low perception of HIV risk among young (pre-sexual) clients, by the decreasing stigma associated with an HIV-positive status in the region, or by the knowledge that HIV care and treatment services were available at the circumcising sites.

### Safety

AE rates fell below those of normal service delivery (from just under 2% to 1%) during the campaign period [13]. This suggests that VMMC providers might become more proficient during high-volume campaigns, although increased oversight and supervision, training by highly qualified VMMC mentors, and quality improvement exercises undertaken during the campaign period may also have helped reduce AE rates.

## Challenges

This case study highlights several major challenges for future high-volume VMMC campaigns. For example, it suggests that ways will need to be found to improve the participation of older male clients. Only 24% of clients served during the Iringa Region campaign were older than 20 years, and previous modeling has shown that for the greatest immediate public health impact, VMMC should cover the sexually active population [10]. Other challenges for future campaigns that are revealed by this case study include the possibility that demand may be higher than anticipated, that there may be insufficient sites providing VMMC services in rural regions, and that the procurement of commodities that are not readily available may require considerable lead times. Furthermore, the case study illustrates how, in many developing countries, surgical instruments, disposable commodities, and pharmaceuticals may need to be imported from abroad, and reusable surgical instruments may have to be transported long distances over difficult terrain to and from facilities with autoclaves. Finally, it draws attention to how infrastructure issues, electricity outages, and the geographic terrain may pose additional challenges in many developing countries.

## Conclusions

The Iringa Region experience shows that VMMC service delivery can be provided to large numbers of men efficiently without compromising quality of service and client safety through a campaign mode of service delivery implemented almost exclusively in the public sector. Although there are considerable challenges associated with implementing such campaigns, they are not insurmountable, as this case study illustrates. Moreover, with contextualization, we suggest that similar campaigns could be replicated in other settings in east and southern Africa where VMMC for HIV prevention has been prioritized.

Notably, since the completion of the Iringa Region campaign, expansion of VMMC services to the remaining five districts of the Iringa Region has become a priority. In December 2010, a three-week campaign that coincided with the school holidays resulted in nearly 3,000 clients being circumcised. By April 2011, all districts in the Iringa Region were offering VMMC and, between June 20 and August 13, 2011, another eight-week campaign served 31,046 VMMC clients across the Iringa Region.

## Abbreviations

AE: adverse event
MCHIP: Maternal and Child Health Integrated Program
PEPFAR: United States President's Emergency Plan for AIDS Relief
STI: sexually transmitted infection
UNAIDS: Joint United Nations Programme on HIV/AIDS
USAID: United States Agency for International Development
VMMC: voluntary medical male circumcision
WHO: World Health Organization
